# Deep Brain Stimulation Imposes Complex Informational Lesions

**DOI:** 10.1371/journal.pone.0074462

**Published:** 2013-08-26

**Authors:** Filippo Agnesi, Allison T. Connolly, Kenneth B. Baker, Jerrold L. Vitek, Matthew D. Johnson

**Affiliations:** 1 Department of Biomedical Engineering, University of Minnesota, Minneapolis, Minnesota, United States of America; 2 Department of Neurology, University of Minnesota, Minneapolis, Minnesota, United States of America; 3 Institute for Translational Neuroscience, University of Minnesota, Minneapolis, Minnesota, United States of America; Hospital Nacional de Parapléjicos, Spain

## Abstract

Deep brain stimulation (DBS) therapy has become an essential tool for treating a range of brain disorders. In the resting state, DBS is known to regularize spike activity in and downstream of the stimulated brain target, which in turn has been hypothesized to create informational lesions. Here, we specifically test this hypothesis using repetitive joint articulations in two non-human Primates while recording single-unit activity in the sensorimotor globus pallidus and motor thalamus before, during, and after DBS in the globus pallidus (GP) GP-DBS resulted in: (1) stimulus-entrained firing patterns in globus pallidus, (2) a monophasic stimulus-entrained firing pattern in motor thalamus, and (3) a complete or partial loss of responsiveness to joint position, velocity, or acceleration in globus pallidus (75%, 12/16 cells) and in the pallidal receiving area of motor thalamus (ventralis lateralis pars *oralis*, VLo) (38%, 21/55 cells). Despite loss of kinematic tuning, cells in the globus pallidus (63%, 10/16 cells) and VLo (84%, 46/55 cells) still responded to one or more aspects of joint movement during GP-DBS. Further, modulated kinematic tuning did not always necessitate modulation in firing patterns (2/12 cells in globus pallidus; 13/23 cells in VLo), and regularized firing patterns did not always correspond to altered responses to joint articulation (3/4 cells in globus pallidus, 11/33 cells in VLo). In this context, DBS therapy appears to function as an amalgam of network modulating and network lesioning therapies.

## Introduction

Chronic pulsatile stimulation of subcortical structures, also known as deep brain stimulation, has become a highly effective surgical therapy for several medication-refractory movement disorders [1,2] and a promising alternative for numerous other brain disorders [3–6]. Building a mechanistic foundation to understand how to modulate neuronal activity to appropriately elicit a mitigating effect on symptoms is critical to the refinement of existing and translation of new surgical targets for DBS therapies. Stimulation pulse train duration, amplitude, location, frequency, and regularity have all been identified as important parameters to both entrain spike activity to the stimuli in and downstream of the DBS target during resting state conditions [7,8] and to generate a therapeutic effect during clinical exam [2,9,10]. The extent to which neuronal firing patterns are modulated by DBS during behavior, and whether the therapeutic effect of DBS is due to suppression [11–16], activation [17–20], or modification of neuronal patterns of activity [21–24] remains unresolved.

Similarities in clinical outcomes between pallidotomy and pallidal DBS therapies led to the formative hypothesis that DBS imposes a virtual lesion of information transmission at the site of stimulation [11]. The virtual lesion is thought to arise through the modulation of neuronal firing rates and patterns in the stimulated nucleus [13,21,25,26] and its efferent targets [7,18–20,27–29], superimposing a pattern of activity with little spike rate variability [30–32] that transmits little or no information – that is, an informational lesion [8,11]. Previous electrophysiological [33,34] and computational [35] studies have provided indirect evidence supporting the informational lesion hypothesis, but these studies have been limited to analysis and modeling of neuronal activity in a ‘resting state’.

**Figure 1:**
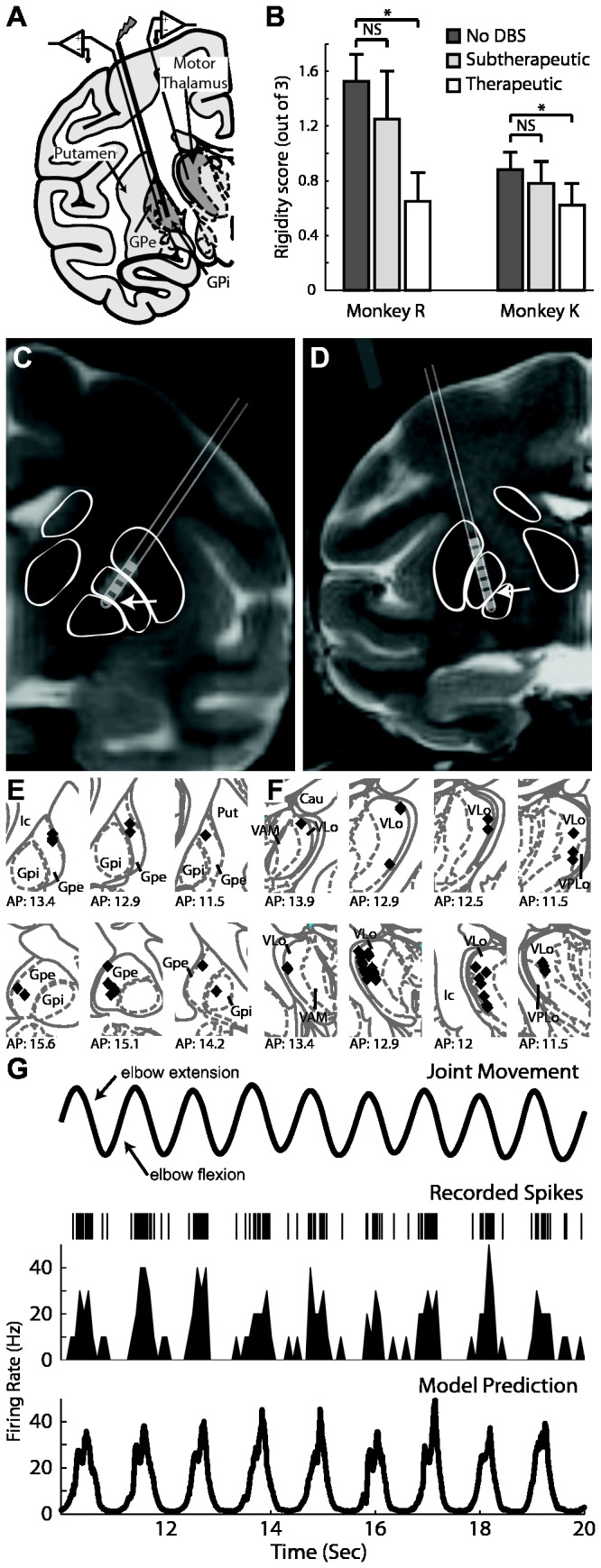
Experimental design used to investigate the effects of GP-DBS on encoding of joint kinematics through the pallidofugal pathway. A: Microelectrode recordings were performed in regions of the globus pallidus and thalamus with spike activity that was responsive to passive joint movement. B: Results of experimenter-blinded muscle rigidity scoring for both monkeys at three DBS settings. C and D: Co-registration of pre-operative MRI and post-electrode implantation CT showing DBS electrode location for monkey R (C) and K (D). E and F: Localization of recorded cells obtained from stereotactic navigation software and overlaid on corresponding atlas plates for monkey R (top) and K (bottom) for both the pallidum (E) and the thalamus (F). G: A generalized linear model (GLM) accounting for position, velocity, and acceleration of the joint movement was applied to determine the correlation between kinematics of the joint movement (top row) and spike activity (2^nd^ row: spike raster, 3^rd^ row: corresponding rate histogram). Bottom row shows the GLM prediction of firing rate.

In this study, passive joint articulation was introduced as a reproducible information signal through the basal ganglia and motor thalamus [36,37] in two non-human primates rendered either parkinsonian or latently dystonic (see *Materials and Methods*, Figure 1). Both received therapeutic benefit on muscle rigidity with pallidal DBS at frequencies of 135 Hz but not 35 Hz [10]. Neuronal spike activity was recorded in the region (globus pallidus) undergoing stimulation at either frequency and in one of its monosynaptic targets (VLo thalamus) to investigate the following questions: 1) In the context of passive joint movement, does DBS modulate neuronal firing patterns similar to those reported in a resting state? 2) Are regularized firing patterns generated by therapeutic DBS necessary to lesion movement-related information? 3) Does DBS produce complete suppression of kinematic information transmission?

## Materials and Methods

### Animals

Two adult rhesus monkeys (*Macaca mulatta*; Monkey R: female, 4.9 kg, 9 yrs old; and Monkey K: male, 11.0 kg, 12 yrs old) were used in this study. All surgical procedures and behavioral protocols were approved by the Institutional Animal Care and Use Committee of the University of Minnesota and complied with United States Public Health Service policy on the humane care and use of laboratory animals. Animals were housed individually with environmental enrichment, provided with water *ad libitum*, and given a range of food options including fresh fruit and vegetables. All efforts were made to provide good care and alleviate unnecessary discomfort, including administration of analgesics prior to and after surgery. Monkey K remains part of a larger study on the physiological mechanisms of GP-DBS. Monkey R was deeply anesthetized with sodium pentobarbital and perfused with a fixative solution containing 4% paraformaldehyde, consistent with the recommendations of the Panel on Euthanasia of the American Veterinary Medical Association.

### Surgical Procedures

Monkey R was rendered moderately parkinsonian with unilateral intracarotid (0.4-0.6 mg/kg) followed by systemic (0.3 mg/kg over 5 days) injections of 1-methyl-4-phenyl-1,2,3,6-tetrahydropyridine) (MPTP) [10,17]. Monkey K was treated with localized unilateral putamenal infusions of 3-nitropropionic acid (3-NP, a compound known to induce dystonic motor signs [38]). The striatal infusions resulted in generally asymptomatic behavior except for persistent, elevated muscle rigidity in its contralateral extremities. In an aseptic procedure under isoflurane anesthesia, animals were instrumented with chambers (Crist instruments, Hagerstown, MD), oriented in the coronal plane (Figure 1A). Microelectrode (impedance 0.5-1 MΩ at 1 kHz) recordings were performed to map the sensorimotor territories within the globus pallidus. A scaled-down version of the human DBS lead (0.75 mm in diameter with 0.5 mm contact height and 0.5 mm spacing between contacts; 4 contacts in monkey R, 8 contacts in monkey K, Numed, Hopkinton, NY) was then implanted such that the lower contacts were located in a region of globus pallidus responsive to passive joint movements. Locations of the electrode contacts along the DBS lead were identified by co-registering post-implant CT images with pre-operative MRI (Figure 1C, D) [10,31].

### Stimulation Parameters

In both animals, monopolar stimulation was found therapeutic for muscle rigidity using an electrode contact at the border of the external (GPe) and internal GPi globus pallidus. DBS settings that reduced muscle rigidity were identified in monkey R as previously described [10,17]. The monopolar stimulation setting that resulted in the largest decrease in muscle rigidity was termed ‘therapeutic’ and used for the remainder of the study (-1 V, 135 Hz, 90 µs pulse width, contact 0) (Figure 1B). In monkey R, this DBS setting also reduced bradykinesia and akinesia [10,17]. In monkey K, the therapeutic DBS setting consisted of monopolar stimulation using an amplitude at 75% of the intensity needed to elicit contralateral muscle contractions (-0.3 mA, 135 Hz, 90 µs pulse width, contact 0). Since monkey K exhibited mild rigidity that was not produced by obvious agonist/antagonist muscles co-contraction, an experimenter blinded to the stimulation protocol manipulated the elbow joint contralateral to the 3-NP infusion and graded rigidity on a modified UPDRS scale ranging from 0-3 (0.88/3 without DBS versus 0.62/3 during high-frequency DBS, p<0.01, Mann–Whitney U test). Subtherapeutic DBS for both animals was delivered through the same contact and pulse width, but at a non-therapeutic intensity in monkey R or at a non-therapeutic frequency (35 Hz) in monkey K.

### Electrophysiology and Motion Capture Recordings

Movement related regions of the globus pallidus and thalamus were identified using firing rates, patterns [39], responses to passive manipulation, and CT-MRI targeting within stereotactic navigation software [40]. To differentiate between VLo and the cerebellar-receiving area of motor thalamus (ventralis lateralis posterior pars *oralis*, VPLo), we performed microstimulation with current intensities between 10 to 60 µA. Neurons were considered to belong to the VPLo if recorded in a region where microstimulation consistently elicited movements with intensities ≤30 µA [41,42]. Microelectrode locations with spike activity were tested for responsiveness to passive manipulation about the contralateral shoulder, elbow, wrist, hip, knee, and ankle. When kinematic-responsive spike activity was encountered, the associated joint was articulated with 30 repetitions before, during and after GP-DBS (Figure 1G). Careful microelectrode targeting into the globus pallidus was necessary to ensure recordings were made of sensorimotor regions while also avoiding collision with the DBS lead. Whenever possible, the response of the recorded unit-spike activity was tested with both therapeutic and sub-therapeutic DBS settings. Joint movements were recorded using motion capture systems (Optotrack, NDI, Waterloo, Ontario for monkey R; Vicon, Centennial, CO, for monkey K) and synchronized with the electrophysiological recordings for movement-triggered analysis.

### Artifact Subtraction Algorithm

A custom template subtraction algorithm similar to the one described previously [43] was used to remove electrical stimulation artifacts from the spike recording data. This procedure reduced the period of recording obscured by stimulation artifacts to a small blanked period (average ~0.5 ms). To prevent biasing the data, similarly blanked regions were introduced in the pre- and post-DBS recording epochs using “virtual stimulation” timestamps at the same stimulation pulse frequency. Template-subtracted recordings were then analyzed in Offline Sorter (Plexon, Dallas, TX) to sort and identify spike activity.

### PSTH and PETH Analysis

Time-stamps of spike activity, stimulation pulses, virtual stimulation pulses, and movement epochs were imported into NeuroExplorer (NeuroExplorer, Littleton, MA). Peri-stimulus time histograms (PSTHs, 0.1-msec bins) were then generated to quantify the entrainment of spike activity to the actual (or virtual) stimulation. Similarly, peri-event timing histograms (PETHs, 50-msec bins) were constructed to assess the relationship between the recorded spike activity and the passive joint articulation. Only cells responding to passive manipulation of one or more joints were included in the analysis; firing rates and PSTHs were calculated only from the recording periods during which passive manipulation was performed. Firing rates calculated before, during, and after DBS were compared for each recorded cell (Mann–Whitney U test, p<0.01, 1-sec bins) and for the population average (Mann–Whitney U test, p<0.01). Modulation of firing patterns by DBS was quantified using the cumulative sum technique for PSTHs [44], with 0.5-msec bins and 99% confidence intervals, which yields a more conservative estimate of changes in firing pattern than by visual inspection. The first millisecond in each PSTH was excluded from the analysis to avoid false positives related to the blanking period from the stimulus subtraction algorithm. This technique considered only cells exhibiting time-locked modulation in their PSTH as statistically significant. If a cell was excited or inhibited, but without time-locked modulation to the stimulation (i.e. a flat PSTH with a different mean from before DBS), the cumulative sum considered the firing pattern of the cell not significantly altered. The overall change in firing pattern was calculated by averaging the population differences between the firing rate during DBS and the firing rate before DBS in each PSTH bin for each recorded cell.

### Generalized Linear Model

A point process model was formulated to quantify the contribution of kinematics to the spiking probability of each neuron. A generalized linear model (GLM) fit function in Matlab (Mathworks, Natick MA) was applied, with Δt=1 ms and covariates of position, velocity, and acceleration in the plane of the tracked limb’s movement (Figure 1G). A neuron was considered responsive to an aspect of movement (position, velocity, or acceleration) if the corresponding parameter for the model was found with a p<0.05. Models were fit to the data obtained before DBS and during DBS to evaluate changes in the kinematic tuning of spike activity as a result of pallidal stimulation. Recorded cells were grouped according to the aspect of movement to which they responded (only to position, velocity, acceleration, or a combination of the three) as identified by the GLM. Within each group, the number of cells that maintained the same responsiveness to movement before and during DBS was tabulated.

## Results

For both nuclei we compared the firing rates before DBS (p=0.011 GP, p=0.32 VLo) and the change in firing rates during DBS (p=0.35 GP, p=0.73 VLo) between the two animal and found no significant difference with a Mann-Whitney test at p<0.01. Cells recorded from the two primates were thus pooled together for further analysis.

### Responses to passive manipulation

The GLM identified 16 cells in globus pallidus and 55 cells in VLo as responsive to joint movement position, velocity, and/or acceleration in the DBS-OFF state (Table 1). In globus pallidus, 11/16 cells encoded joint position, 14/16 encoded velocity, and 7/16 encoded acceleration with most (12/16) encoding multiple aspects of the joint movement. Similarly, of the cells recorded in VLo, 37/55 encoded position, 46/55 encoded velocity, and 13/55 encoded acceleration with 33/55 encoding more than one aspect of movement. In contrast to the globus pallidus where 20% of the cells were responsive to a single aspect of movement (i.e. position, velocity, or acceleration), 40% (22/55 cells) of cells in the VLo were tuned to one kinematic aspect of movement.

**Table 1:** Neurons that retained all/any of their tuning to joint movement during DBS.

**Target**	**DBS**	**P**	**V**	**A**	**P+V**	**P+A**	**V+A**	**P+V+A**	**Total**
Globus	Effective	*1/1 (1)*	*1/1 (3)*	*−*	*0/3 (5)*	*0/1 (1)*	*1/1 (2)*	*1/3 (4)*	4/10 (16)
Pallidus	Ineffective	*1/1 (2)*	*1/1 (1)*	*−*	*2/4 (4)*	*2/2 (2)*	*−*	*1/1 (1)*	7/9 (10)
VLo	Effective	*4/4 (7)*	*11/11 (14)*	*1/1 (1)*	*14/19 (21)*	*0/1 (1)*	*2/3 (3)*	*2/7 (8)*	34/46 (55)
Thalamus	Ineffective	*1/1 (1)*	*5/5 (6)*	*−*	*16/19 (20)*	*0/1 (1)*	*1/5 (5)*	*1/4 (7)*	24/35 (40)

Note: total number of recorded neurons in parentheses; P: position, V: velocity, A: acceleration

### Pallidal responses to GP-DBS

**Figure 2:**
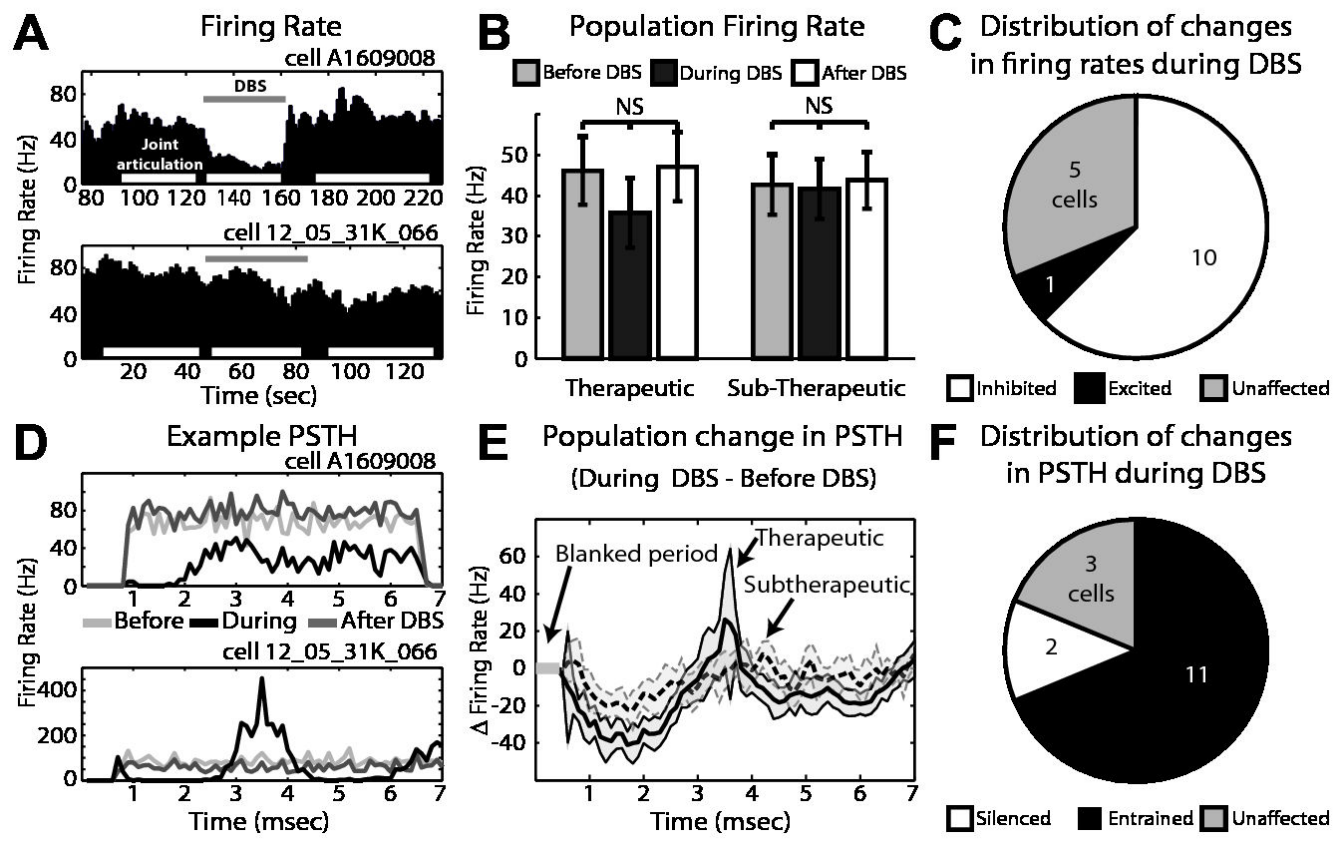
Cellular responses in globus pallidus to GP-DBS during joint movement. A: Example of firing rate in two pallidal cells before, during (grey bar), and after DBS. Periods of joint articulation used for analysis are denoted by white bars. B: Population average firing rate change during therapeutic and sub-therapeutic DBS. Error bars indicate +/- 1 SEM (n=16 therapeutic DBS, n=10 sub-therapeutic DBS). C: Proportion of recorded cells with statistically significant changes in firing rate during therapeutic DBS. D: Corresponding PSTHs to the example pallidal neurons shown in part A, before (light grey), during (black) and after DBS (dark grey). E: Population average change in firing pattern during therapeutic (dark grey) and subtherapeutic (light grey - dashed) DBS. Filled areas indicate +/- 1 SEM. F: Proportion of recorded cells with statistically significant changes in their PSTHs during therapeutic DBS.

During passive joint movement, microelectrode recordings were performed before, during, and after therapeutic (n=16 cells) and sub-therapeutic (n=10 cells) GP-DBS in the globus pallidus. The average firing rate across all cells decreased during therapeutic DBS (from 46.6 ± 7.9 to 34.5 ± 8.1 Hz), though the change was not statistically significant (p=0.26, Mann–Whitney U test), and remained unchanged during sub-therapeutic stimulation (42.7 ± 7.4 to 41.7 ± 7.4 Hz, p=0.79) (Figure 2A,B). The overall decrease in the population average firing rate with therapeutic DBS was attributed to significant inhibition in 63% of the recorded cells, with an additional 6% showing excitation and 31% showing no significant alteration in firing rate (Figure 2C). Therapeutic DBS also induced a population-wide firing pattern change (Figure 2D,E), consisting of an early inhibitory period (0.5-3 ms) followed by an excitatory period (3-4 ms) and a second inhibitory period (4-6.5 ms) (Figure 2E). This triphasic stimulus-entrained firing pattern was observed in 6/16 cells, with another 5/16 cells exhibiting a single inhibitory phase (0.5-3 ms) after stimulus delivery (Figure 2F). Sub-therapeutic DBS altered firing patterns in a smaller fraction of the recorded population (30%), each of which showed morphologically similar firing pattern changes to therapeutic DBS.

### Thalamic responses to GP-DBS

**Figure 3:**
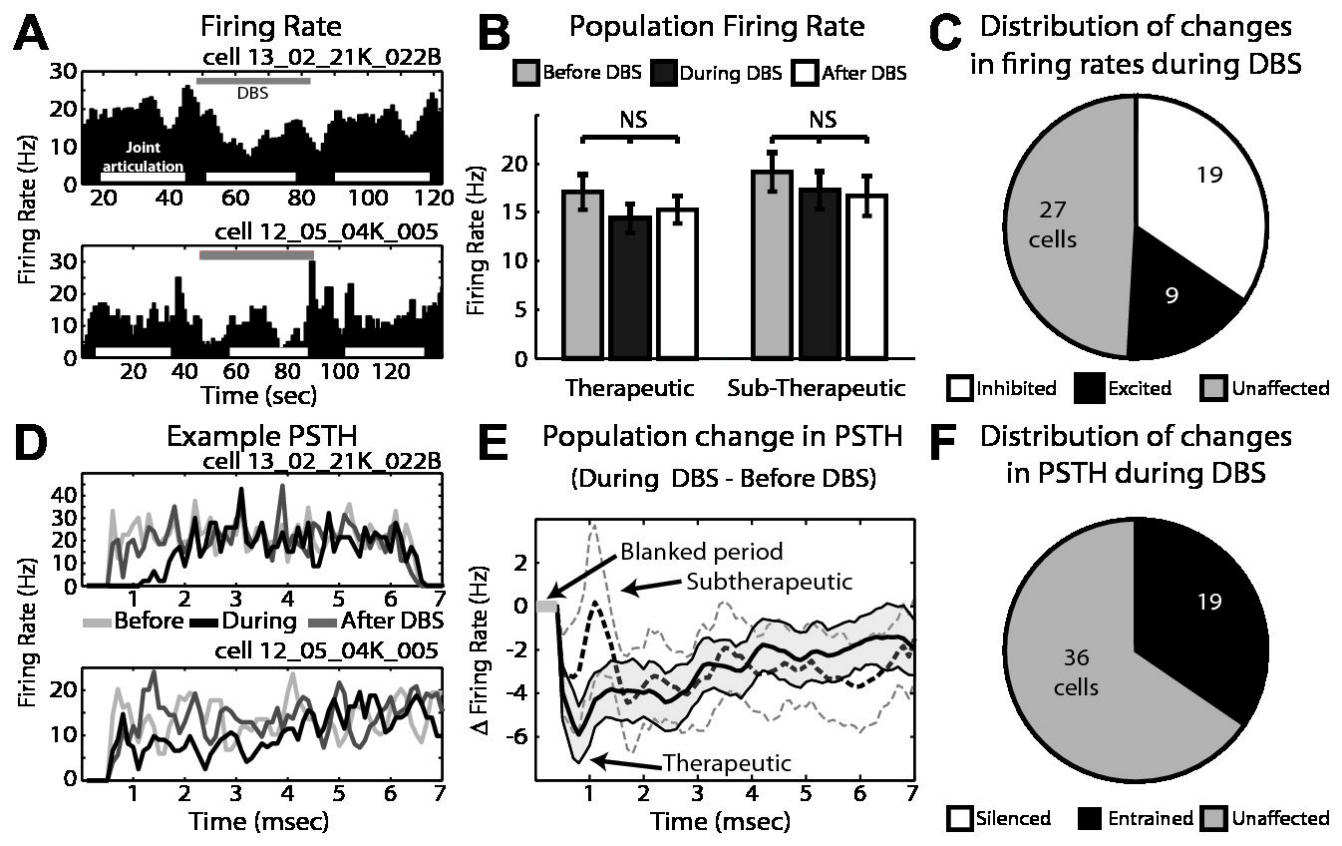
Cellular responses in VLo thalamus to GP-DBS during joint movement. A: Example of firing rate in two VLo cells before, during (grey bar), and after DBS. Periods of joint articulation used for analysis are denoted by white bars. B: Population average firing rate change during therapeutic and sub-therapeutic DBS. Error bars indicate +/- 1 SEM (n=55 therapeutic DBS, n=40 sub-therapeutic DBS). C: Proportion of recorded cells with statistically significant changes in firing rate during therapeutic DBS. D: Corresponding PSTHs to the example VLo neurons shown in part A, before (light grey), during (black) and after DBS (dark grey). E: Population average change in firing pattern during therapeutic (dark grey) and subtherapeutic (light grey - dashed) DBS. Filled areas indicate +/- 1 SEM. F: Proportion of recorded cells with statistically significant changes in their PSTHs during therapeutic DBS.

Also during passive joint movement, microelectrode recordings were performed in the pallidal-receiving area of motor thalamus (VLo) before, during, and after therapeutic (n=55) and sub-therapeutic (n=40) GP-DBS. The average firing rate of the population was not changed during therapeutic (from 17.8 ± 1.8 to 14.4 ± 1.5 Hz, p=0.25) or sub-therapeutic DBS (from 19.1 ± 2 to 17.3 ± 1.9 Hz, p=0.39) (Figure 3A,B). However, a substantial fraction of cells exhibited significant changes in firing rates, including inhibition (34.5%, 19/55 cells) and excitation (16%, 9/55 cells) that balanced each other in terms of the population’s overall firing rate (Figure 3C). GP-DBS was found to modulate firing patterns of VLo neurons, with the population average firing pattern consisting of an initial inhibitory phase (0.5-3 ms) (Figure 3D,E). Based upon the PSTH analysis, 35% (19/55) of cells were entrained to therapeutic DBS (see Figure 3F). During sub-therapeutic DBS 12.5% (5/40) displayed a significantly altered PSTH pattern of firing with changes morphologically similar to those seen during therapeutic stimulation.

### Effect of GP-DBS on pallidal responses to joint kinematics

**Figure 4:**
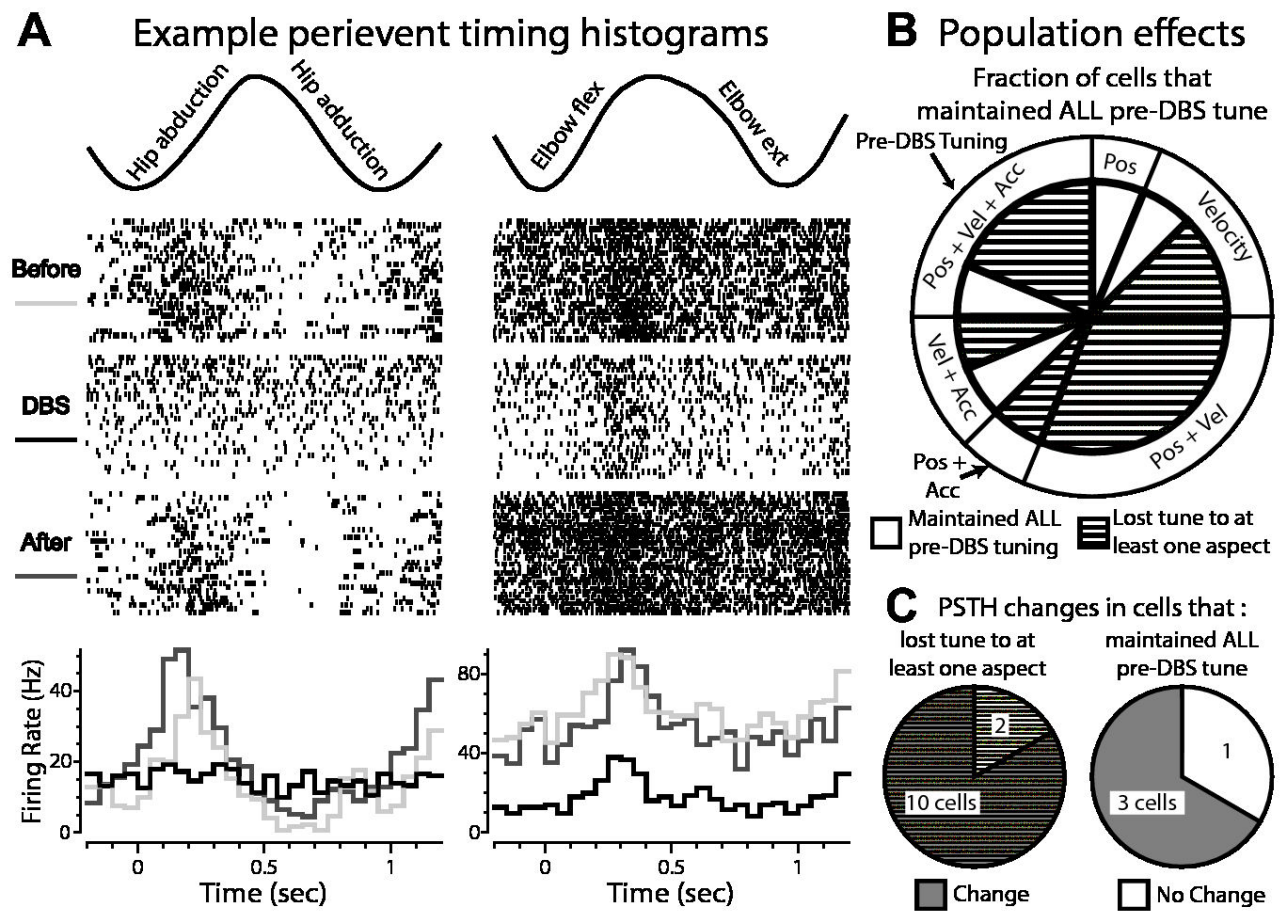
Effect of GP-DBS on kinematic tuning of globus pallidus spike activity. A: Two examples of modulated responses to joint movement during therapeutic DBS (top: motion capture data of the joint movement; middle: corresponding raster plots triggered to the beginning of each movement cycle; bottom: peri-event time histograms showing responses before, during, and after DBS). B: Population analysis of cells that did and did not maintain tuning to joint movement during therapeutic DBS. Outer pie chart shows the proportion of the recorded population tuned in the DBS-OFF condition to aspects of the joint movement (i.e. position, velocity, acceleration, or a combination). Inner pie chart shows the fraction of cells in each group that maintained tuning during DBS (white), or lost some aspect of tuning during therapeutic DBS (hashed). C: (left) Proportion of the recorded population with partial or complete loss of tuning during therapeutic DBS in which the accompanying PSTH was also modulated (grey hash) or unchanged (white hash); (right) proportion that maintained tuning during therapeutic DBS and whose PSTH was modulated (grey) or unchanged (white) by therapeutic DBS.

**Figure 5:**
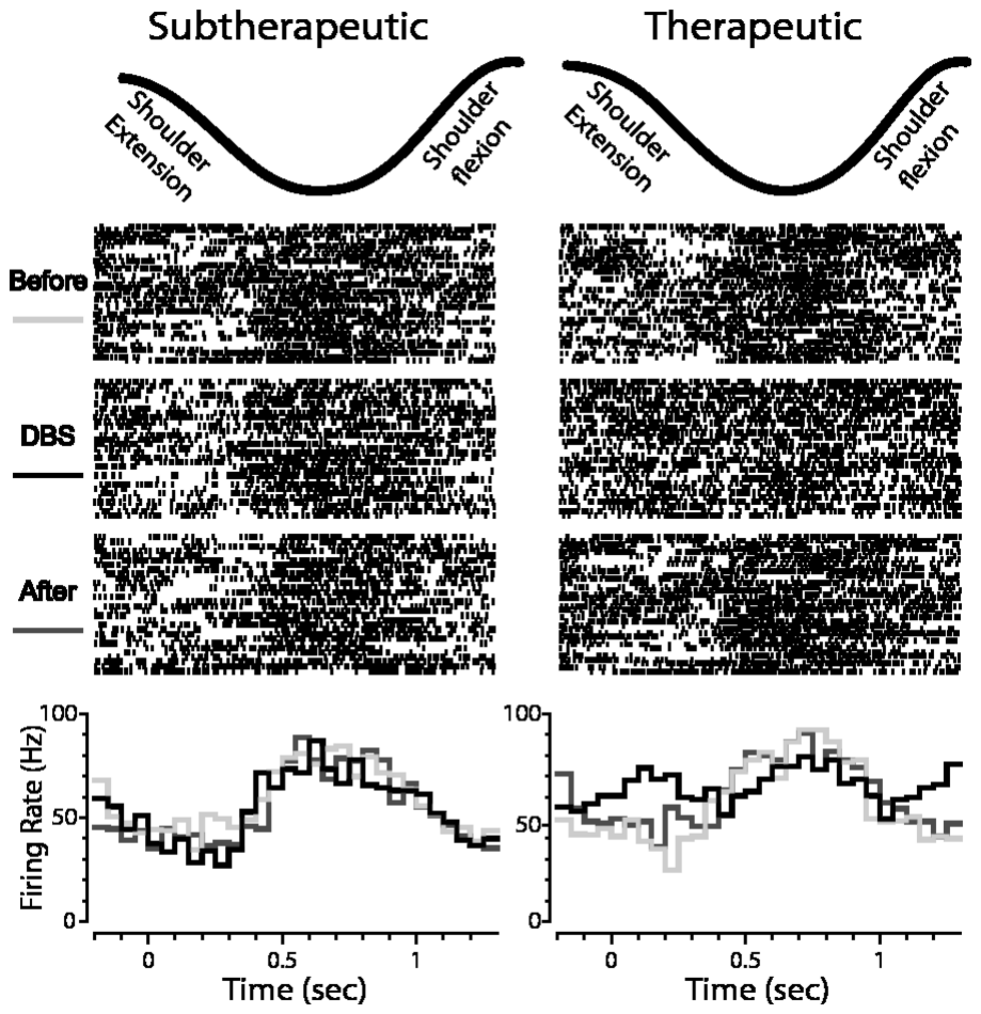
Neuronal encoding of joint movement during subtherapeutic and therapeutic DBS in globus pallidus. Shown is an example of the response of a cell to shoulder flexion/extension before, during and after subtherapeutic DBS (left) and therapeutic DBS (right) (top: motion capture data of the joint movement; middle: corresponding raster plots triggered to the beginning of each movement cycle; bottom: PETHs showing responses before, during, and after DBS).

Comparisons of peri-event time histograms before, during, and after stimulation revealed a variety of changes in tuning, including broadened tuning, loss of tuning, and maintenance of tuning with decreased background activity in the globus pallidus (Figure 4A). Generalized linear model analysis of the recording data demonstrated that the majority of pallidal cells (75%, 12/16) lost responsiveness to at least one aspect of joint kinematics during therapeutic DBS (Figure 4B). This loss of tuning occurred regardless of whether the cells were tuned to position, velocity, acceleration, or a combination of the three aspects (Table 1). During sub-therapeutic DBS, however, the majority of pallidal cells (70%, 7/10) maintained their pre-DBS tuning to movement such as shown in Figure 5. We found that 85% (10/12) of cells in globus pallidus that exhibited a complete (5/6) or partial (5/6) loss of responsiveness to passive joint movement during therapeutic DBS also exhibited significant modulation in their PSTH (Figure 4C). At the same time, 75% (3/4) of cells that maintained their tuning to passive joint movement during therapeutic DBS nevertheless exhibited significant changes in their PSTH during therapeutic DBS (Figure 4C).

### Effect of GP-DBS on motor thalamic responses to joint kinematics

**Figure 6:**
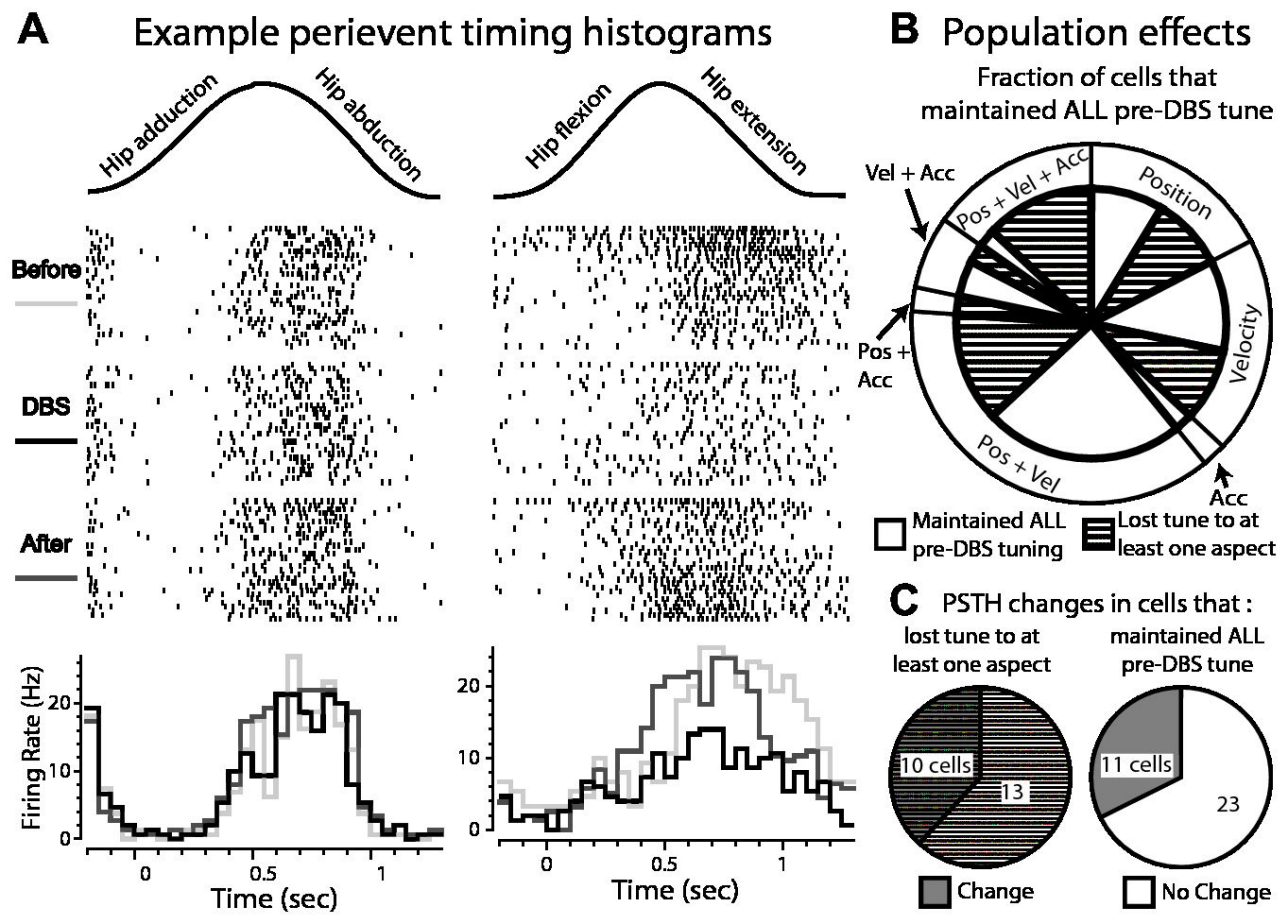
Effect of GP-DBS on kinematic tuning of VLo spike activity. A: Two examples of responses to joint movement during therapeutic DBS. B: Population analysis of cells that did and did not maintain tuning to joint movement during therapeutic DBS. C: (left) Proportion of the recorded population with partial or complete loss of tuning during therapeutic DBS whose PSTH was also modulated (grey hash) or unchanged (white hash); (right) Proportion that maintained tuning during therapeutic DBS and whose PSTH was modulated (grey) or unchanged (white) by therapeutic DBS.

DBS also altered responsiveness to joint kinematics in VLo thalamus, albeit to a lesser extent than that found in the globus pallidus (Figure 6A). In VLo, 38% (21/55) of cells exhibited loss of tuning to at least one kinematic aspect during therapeutic GP-DBS (Figure 6B). Of these, only 39% (8/21 cells) displayed concurrent entrainment of their PSTH during therapeutic DBS (Figure 6C). Of the cells that maintained their kinematic tuning during therapeutic DBS, 32% (11/34 cells) were found to have significant alterations in their PSTH (Figure 6C). In contrast to recordings in the globus pallidus, the fraction of cells which lost tuning to at least one aspect of movement was comparable between therapeutic (38%, 21/55) and sub-therapeutic stimulation (40%, 16/40).

## Discussion

This study, which directly tested the informational lesion hypothesis of DBS, quantified the effects of GP-DBS on firing rate, firing pattern, and tuning to passive joint movement through the pallidofugal pathway. Spike recordings were collected in the sensorimotor areas of the globus pallidus and thalamus from two non-human primates, both of whom received improvement in muscle rigidity with GP-DBS. The coupled spike recording and joint movement data supported the hypothesis that regularized firing patterns created partial informational lesions since spike activity along the pallidofugal pathway still encoded some aspect of joint movement during therapeutic DBS despite exhibiting more regular firing patterns.

### DBS modulates firing rates and patterns within and downstream of the stimulated target

During joint articulation, the proportion of pallidal cells with modulated firing rates (10/16 inhibited, 1/16 excited, 5/16 no change) during therapeutic GP-DBS was consistent with previous resting state electrophysiology studies in humans [13,45] and non-human primates [12,18,25,26]. Previous resting state studies have reported a broad range of overall firing rate changes in globus pallidus with GP-DBS, ranging from no effect [26], to a comparable decrease (18.7% [12]), to stronger inhibition (60% [25]) in the resting state. These results, however, should be interpreted in the context of several differences in experimental preparation that include dimensions of the stimulating electrode(s) [46], location of the active electrode(s) within GP [32], amplitude of the stimulation [13,32,45,47], and proximity of the recording microelectrode to the stimulated DBS contact(s) [13,45].

We also observed that a proportion of VLo cells inhibited (27/55, 34.5%) and excited (9/55, 16%) during GP-DBS to be comparable to those observed previously in resting state conditions (45% inhibited versus 16% excited by [18]; 48% versus 8% by [20]). The overall firing rate in VLo thalamus during GP-DBS did not decrease as strongly as previous studies, however. This finding may stem from a previous observation that motor thalamic firing rates during a movement task were not inhibited as greatly by GP-DBS as during resting state conditions [18]. Moreover, if the spatial volume of modulation within GP inhibits somatic activity more than drives GPi efferent activity along the pallidofugal pathway [32], only mild inhibition in VLo will result.

Also consistent with previous studies, we observed that the majority of recorded neurons in the sensorimotor globus pallidus (11/16 cells) developed a more regular pattern of activity during GP-DBS [25,26,45]. The firing pattern became time-locked to the stimulation with inter-stimulus spike activity that consisted of an early inhibitory phase for most of the recorded population, which in some cases was followed by a middle excitatory phase, and a late inhibitory phase. In comparison to the strong regularity observed in the globus pallidus, a smaller subset of neurons in the VLo (19/36 cells) displayed altered firing patterns during GP-DBS. Thus, the modulation of firing patterns in VLo thalamus reinforces the notion that GP-DBS produces network-level effects throughout the motor thalamus [20,28] and motor cortices [17].

### GP-DBS induces a partial information lesion in globus pallidus and thalamus

DBS is thought to substitute the output of the stimulated nucleus with a regularized, high frequency pattern of activity devoid of meaningful physiological content [7,18,28,48]. Such an “informational lesion” is thought to explain the similarity in clinical outcomes between surgical lesion and DBS procedures [11]. According to this hypothesis, the high frequency inter-stimulus pattern of activity created by DBS, likely low-pass filtered by intrinsic synaptic properties, imposes a more regular pattern of activity in downstream nuclei that is time-locked to the stimulation and interferes with transmission of information through the stimulated pathway. While computational modeling studies and electrophysiological analysis of resting state activity have been used to investigate this theory, the present study provides the first experimental evidence to probe the informational lesion hypothesis of DBS in the context of sensorimotor input.

If DBS created a complete informational lesion, one would expect a total loss of correlation between the firing rate of a neuron and the aspects of movement encoded by the neuron. At the same time, one may expect a suppression of information in the nuclei downstream of stimulation, assuming DBS drives axons projecting from the stimulated brain region [30,32]. Passive joint manipulation was introduced in this study as a reproducible physiological signal known to be transmitted and processed within the basal ganglia-thalamic circuit. Previous studies have shown that the functional topography present in the motor cortices is conserved throughout the basal ganglia and motor areas of thalamus [36], where cells respond to passive joint movement about the contralateral side of the body [37,42,49]. The neuronal response to passive joint manipulation in the basal ganglia-thalamic circuit represents not only a physiologically relevant signal that can be used to evaluate the impact of DBS on information transmission, but also an aspect of neuronal coding known to be altered in movement disorders such as Parkinson’s disease [50,51] and dystonia [52–54].

Modeling studies suggest GP-DBS has different effects on spike activity between somata versus efferent and afferent axons within the globus pallidus (10 [32]. Under this model, activation of efferent axons would produce antidromic collision with naturally occurring spikes, while activation of afferent axons and somata in the globus pallidus would produce time-locked alteration of somatic activity. In both cases, information passing through the stimulated area could be lost or improperly processed due to GP-DBS. In accordance with the hypothesis that DBS generates an informational lesion within the stimulated area, we found that during GP-DBS the majority of pallidal cells were unable to encode movement-related information with the same fidelity as prior to GP-DBS. Surprisingly, however, during GP-DBS, 6/16 pallidal cells were still able to encode aspects of passive joint movement faithfully, and an additional 4/16 pallidal cells fully retained their movement encoding. Given the relatively small number of cells studied here, caution must be used when drawing general conclusions, but the results seem to suggest that the informational lesion produced by DBS may not be complete and that cells in the stimulated target still encode behaviorally relevant information during therapeutic stimulation.

At the same time, tonic activation of efferent fibers will induce a non-physiological, high frequency, regular GABAergic input to downstream nuclei, potentially interfering with information processing and transfer in the VLo, thus “spreading” the informational lesion to areas distal to stimulation. However, in contrast to globus pallidus, more than twice as many cells in the VLo were able to retain their original tuning during GP-DBS (60% in motor thalamus versus 25% in globus pallidus), suggesting that the partial loss of tuning observed in GP did not proportionally transfer to the VLo thalamus. While it is possible that GP-DBS attenuated the synaptic strength of pallidal projections within thalamus, as it was found to induce inhibitory synaptic plasticity within the GP [55], such decreased responsiveness would be expected to occur at longer time scales of stimulation than those investigated in this study. It is also possible that given the stable condition of the pathophysiology in both non-human primates, motor thalamus may already have been in a state that is less responsive to basal ganglia input. Additionally, the globus pallidus constitutes only one of several inputs to VLo thalamus [56], and these inputs including extensive innervation from corticothalamic projections may still send kinematic information to VLo despite modulation through the pallidofugal pathway.

### Regularization of firing patterns is not sufficient to modulate movement-related information

Surprisingly, many of the recorded cells that retained their tuning to passive joint movement, displayed a clear alteration in firing pattern during therapeutic DBS (3/4 cells in globus pallidus and 11/33 in VLo). This finding suggests that even when neuronal activity is entrained to DBS, the stimulation pulse train does not completely override the processing of relevant behavioral information. At the same time, a portion of the recorded neuronal population that exhibited alteration in spike activity during passive limb movements showed no significant modulation in their firing pattern or rate during therapeutic DBS (2/12 in globus pallidus, 13/23 in VLo). Therefore, the mechanism of loss of tuning cannot be fully explained by the direct effect of electrical stimulation on the recorded neuron, suggesting that an indirect network mechanism may be involved.

## Conclusions

This study showed that the physiological mechanisms of DBS in the motor basal ganglia-thalamocortical network are similar but not equivalent to a complete informational lesion. Neurons in the nucleus of stimulation and in downstream targets can lose or alter tuning to movement, and their firing patterns can be entrained by DBS, but the two phenomena are not necessarily co-dependent. As such the data support a mechanism for DBS in which stimulation modulates information transmission but that such an effect is not completely inhibitory to information transmission.
